# Association between the Number of Days/Week of Different Levels of Physical Activity and Chronic Pain in People of Different Races: A Mendelian Randomization Study

**DOI:** 10.3390/jpm14010050

**Published:** 2023-12-29

**Authors:** Sumei Luo, Minjing Yang, Haojun Yang, Qulian Guo, Yunjiao Wang, E Wang

**Affiliations:** 1 Department of Anesthesiology, Xiangya Hospital, Central South University, Changsha 410008, China; luosumei@csu.edu.cn (S.L.); 208107037@csu.edu.cn (M.Y.); 2204150323@csu.edu.cn (H.Y.); qulianguo@csu.edu.cn (Q.G.); 2 Clinical Research Center of Hunan Province for Anesthesia and Perioperative Medicine, Changsha 410008, China; 3 National Clinical Research Center for Geriatric Disorders, Xiangya Hospital, Central South University, Changsha 410008, China

**Keywords:** chronic pain, physical activity, Mendelian randomization, race, genome-wide association study

## Abstract

Objective: Regular physical activity is beneficial for health, but the effect of the number of days/week of physical activity on chronic pain (CP) remains unclear, so we used a two-sample Mendelian randomization (MR) analysis to explore the relationship between the number of days/weeks of different levels of physical activity and chronic pain in people of different races. Methods: We obtained summary data from genome-wide association studies (GWASs) on the number of days/week of physical activity and multisite chronic pain in European, South Asian, East Asian, Middle Eastern, and African American populations. The single-nucleotide polymorphisms (SNPs) of the exposed data were visualized with a Manhattan plot via the R program. MR analysis was performed by the MR-Base platform. Results: The results indicated that a higher number of days/week with ≥10 min of walking protects against CP in African American and Afro-Caribbean populations (inverse-variance weighting, IVW *p* < 0.05) but has little effect on people of different races (IVW *p* > 0.05). A higher number of days/week with ≥10 min of moderate physical activity increased the risk of CP in European and South Asia (IVW *p* < 0.05) but had little effect on people of different races (IVW *p* > 0.05). The number of days/week of ≥10 min of vigorous physical activity increased the risk of CP in Europeans (IVW *p* < 0.05) and protected against CP in African Americans and Afro-Caribbeans (IVW *p* < 0.05). Conclusions: A higher number of days/week of moderate and vigorous physical activity increased the risk of CP in Europeans; however, a higher number of days/week of walking and vigorous physical activity may protect against CP in African American and Afro-Caribbean individuals.

## 1. Introduction

Chronic pain (CP) refers to persistent or intermittent pain that lasts for more than 3 to 6 months, influencing approximately 20% of people worldwide [1]. Chronic regional pain accounts for 11.1% of chronic pain, chronic back pain accounts for 10.1%, and leg and foot pain accounts for 7.1% [2]. Most patients with chronic pain typically suffer from more than one type of pain [3]. Chronic pain can lead to a significant decrease in quality of life, resulting in the onset of chronic diseases and mental illnesses such as depression, anxiety, and drug abuse disorders. Suicidal ideation in patients with chronic pain can even reach up to 20% [4]. Considering that CP is an important disease affecting millions of people and an important public health problem, with a high incidence rate and mortality, modifiable risk factors that could lower the incidence of CP must be identified.

The modifiable risk factors for CP include smoking, alcohol, obesity, nutrition, sleep problems, employment status and occupational factors, sunshine and vitamin D, and physical activity/exercise [5]. Previous studies on the relationship between physical activity and CP have different results, with some suggesting that physical activity reduces the risk of CP [6,7,8], some suggesting that physical activity increases the risk of CP [9,10], and some suggesting that physical activity has no effect on CP [11,12]. This different outcome may be due to factors such as gender, race, socioeconomic status, alcohol consumption, sleep, and depression that influence the impact of physical activity on CP. Different sensitivities to pain stimuli result in varying perceptions of pain among individuals of different genders and races [13,14]. Socioeconomic status can lead to differences in professions, thereby affecting the incidence of CP. Fatoye’s research showed that there was a higher prevalence of low back pain in high-income countries than low-middle income countries [15]. Alcohol consumption can cause deficiency in the activity of the endogenous opioid peptide system and lowers the pain threshold [16]. Kastelic’s research suggests that additional sleep can increase the risk of low back pain, and the risk of low back pain is completely different between light-intensity physical activity and moderate- to vigorous-intensity physical activity [17]. The inflammatory process of depression induces changes in central nervous system immune regulation, which may lead to pain sensitization and chronicity [18]. However, it is worth noting that biological and social factors of race play a crucial role in the lives of individuals [19], and it can have an impact on CP alone or on other factors to play a role in CP. There is currently no research analyzing the impact of different levels of physical activity on chronic pain in different ethnic groups. Therefore, to accurately assess CP in different ethnic groups, we analyzed the relationship between different degrees of physical activity and CP in people of different races.

In the present study, we used Mendelian randomization to assess the effects of the number of days/week of physical activity on CP in people of different races. Participants included individuals from European, South Asian, East Asian, Middle Eastern, and African American populations. The categories of physical activity included the number of days/week with ≥10 min of walking, ≥10 min of moderate physical activity, ≥10 min of vigorous physical activity. The results of this study may provide evidence for effective prevention of CP, identify high-risk populations for CP, and provide ideas for developing new therapies for CP.

## 2. Methods

### 2.1. Study Design

**Figure 1:**
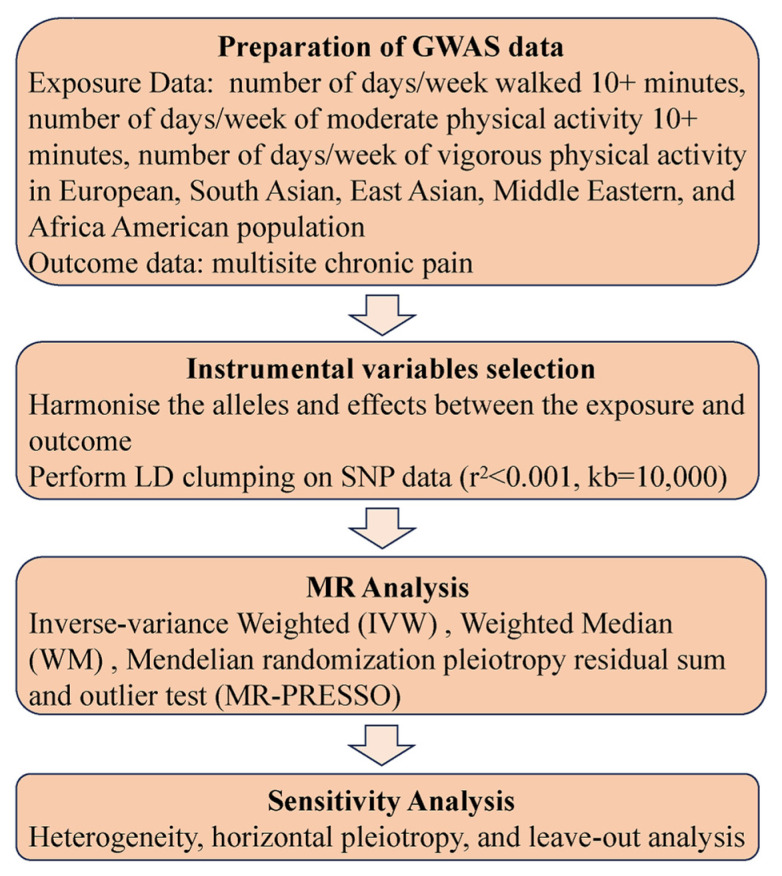
Flowchart of the MR analysis.

The present study utilized bidirectional two-sample MR analyses to examine the association between physical activity and CP based on summary statistics from a Genome-Wide Association Study (GWAS). This study used publicly available aggregated statistical data and therefore did not require ethical approval. The flowchart is shown in Figure 1.

### 2.2. Physical Activity Samples

In this study, we considered the number of days/week of three different categories of >10 min of physical activity in European, South Asian, East Asian, Middle Eastern, and African American populations based on previous research, including walking, moderate physical activity, and vigorous physical activity. The summary statistics for the number of different physical activities over 10 min were sourced from the UK Biobank Consortium, which is a large-scale biomedical database of GWASs. Details of the GWASs used in this study are provided in Table 1.

### 2.3. CP Samples

The GWAS data for CP were obtained from ukb-e-MCP-AFR, which was sourced from the UK Biobank Consortium, and we chose it because it is the dataset with the most SNPs in the search results for CP, including 6195 African Americans and African Caribbeans and 15,534,171 SNPs.

### 2.4. MR Analysis

First, we visualized the SNPs of the exposed data in the Manhattan plot via the package “CMplot” of the R program (version 4.2.3, R language, Auckland, New Zealand). Further MR analysis was performed by the package “TwoSampleMR” of the R program (version 4.0.3, R language, Auckland, New Zealand) in the MR-Base platform, and the APP version was 1.4.3 8a77eb (25 October 2020). We used the inverse-variance weighting (IVW) method, weighted median estimator, and MR-Egger regression to investigate the causal relationship between the number of physical activities and CP [20,21]. We identified the SNPs associated with physical activity by setting the threshold of statistical significance to *p* < 5 × 10^−5^, LD r^2^ < 0.001, and F value > 10. A causal effect of exposure on the increase in the risk of outcome was considered significant if *p* < 0.05.

### 2.5. Sensitivity Analysis

The sensitivity of the results was determined by the leave-one-out method by removing single SNPs one by one and calculating the effect of the remaining SNPs by the IVW method. In this way, we examined the effects of individual SNPs on causality.

## 3. Results

### 3.1. Genetic Instruments for the Number of Physical Activities in People of Different Races

**Figure 2:**
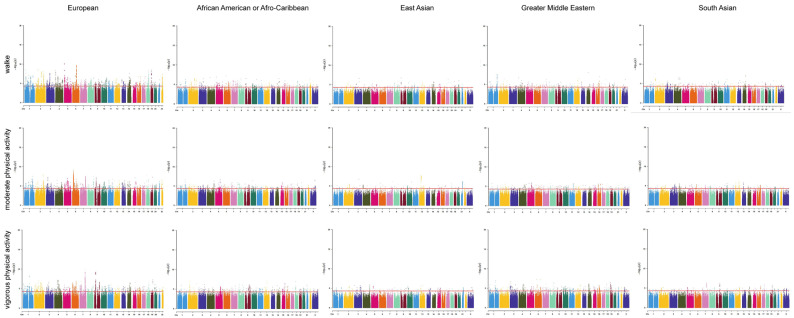
Manhattan plot depicting the SNPs associated with the number of days/week with ≥10 min of various categories of physical activity. The *X*-axis is arranged from chromosome 1 to chromosome X, and the position on each chromosome is represented by dots. The *Y*-axis represents the results of the association analysis, represented by −log10 (*p*-value), with higher points indicating genes or SNPs with more significant associations. The red line parallel to the *X*-axis is the threshold line for *p*-values, and points above this line indicate that these loci represent significantly associated genes or SNPs.

We generated Manhattan plots for the SNPs in the number of physical activities using the “CMplot” R package (Figure 2). Our study included the number of days/week with ≥10 min of walking, ≥10 min of moderate physical activity, and ≥10 min of vigorous physical activity, and European, African American, Afro-Caribbean, East Asian, Greater Middle Eastern, and South Asian populations were represented in every physical activity category. The *p* values for the number of days/week of each physical activity category were 5 × 10^−5^ in the Manhattan plot. The dots above the red line represent the SNPs we screened in the physical activity samples, and it can be seen that each physical activity category is associated with many different SNPs, with the physical activity categories of Europeans having the highest number of different SNPs. The detailed information of identified SNPs is illustrated in Table S1.

### 3.2. Causal Effects of the Number of Physical Activities on CP in People of Different Races

**Figure 3:**
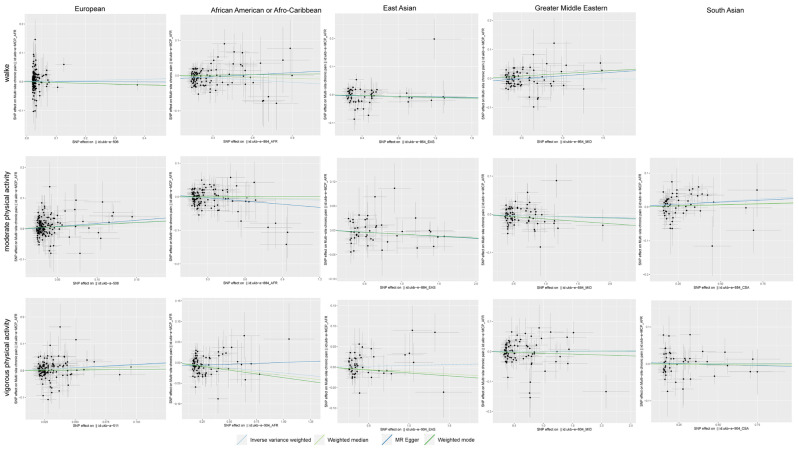
Scatter plots depicting the results of Mendelian randomization (MR) analyses investigating the associations between the number of days/week with ≥10 min of various categories of physical activity and CP. The light blue line represents the inverse-variance weighted estimate, the light green line represents the weighted median estimate, the dark blue line represents the Mendelian randomization–Egger estimate, the dark green represents the weighted mode estimate. The *x*-axis values represent the effect of SNPs on the physical activity. The *y*-axis values represent the effect of the SNPs on CP.

Causal effects of the number of physical activities on CP in people of different races using different Mendelian randomization (MR) methods are provided in Table 2. Regarding the effect of the number days/week with ≥10 min of walking on CP, we observed a significant protective effect on African Americans and Afro-Caribbean individuals (OR_IVW_ = 0.979; 95% CI 0.959, 1.000, *p* = 0.045). No significant association was observed between the number of days/week with ≥10 min of walking and CP in people of different races. The results showed that the number of days/week of ≥10 min of moderate physical activity was associated with an increased risk of CP in Europeans (OR_IVW_ = 1.149; 95% CI 1.052, 1.256, *p* = 0.002) and South Asians (OR_IVW_ = 1.032; 95% CI 1.009, 1.056, *p* = 0.007). However, no significant association between the number of days/week of ≥10 min of moderate physical activity and CP was observed in people of different races. In the number of days/week of ≥10 min of vigorous physical activity on CP, we observed a significant causal effect on Europeans (OR_IVW_ = 1.243; 95% CI 1.090, 1.416, *p* = 0.001) and a protective effect on African Americans and Afro-Caribbeans (OR_IVW_ = 0.975; 95% CI 0.957, 0.994, *p* = 0.010). However, there was no significant association between the number of days/week of ≥10 min of vigorous physical activity and CP in people of different races. The main results of our study are visualized by scatter plots (Figure 3).

Heterogeneity and pleiotropy analysis of the exposure data are shown in Table 3. The heterogeneity *p* value of most exposure data was above 0.05, except for Ukb-e-904-MID and Ukb-e-904-CSA, suggesting that most exposure data in our study have no heterogeneity. For Ukb-e-904-MID and Ukb-e-904-CSA, we used the random-effects IVW model as the major analysis method. In addition, the pleiotropy *p* value of all exposure data was above 0.05, suggesting that there was no potential pleiotropy in our study.

**Figure 4:**
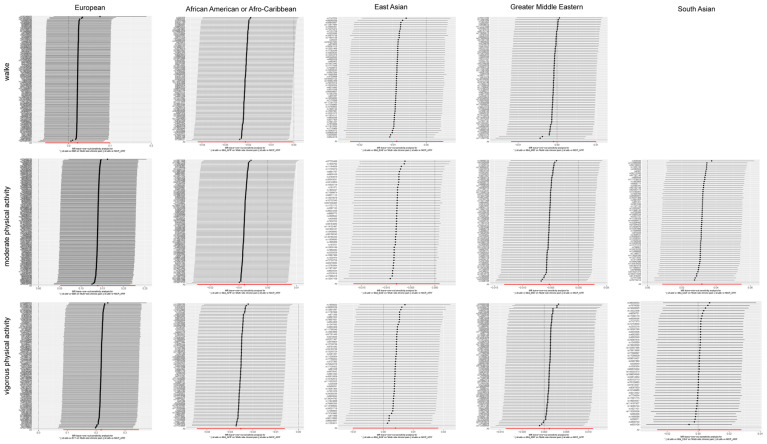
The results of a leave-one-out analysis on Mendelian randomization (MR). Each black line in the figure corresponds to the outcome of the MR analysis when one single nucleotide polymorphism (SNP) was removed from the analysis, while the remaining SNPs are on the left. The error bar represents the 95% confidence interval with the method of inverse-variance weighting.

Moreover, sensitivity analysis of the primary outcome was conducted by leave-one-out analysis, excluding one significant SNP each time. Every significant SNP of physical activity on CP was heterogeneous with other SNPs (Figure 4).

## 4. Discussion

As a worldwide public health concern, chronic pain affects approximately 30% of people [22,23]. Prior research has revealed that increased frequency, duration, and intensity of physical activity are associated with less chronic pain [24,25,26]. There are also studies showing that physical activity could be an important contribution to prevention of chronic pain [27]. In addition, there are also studies that suggest that physical activity is not associated with the incidence of neck and shoulder pain [28]. In conclusion, previous studies have been controversial regarding the relationship between physical activity and CP, and the connection between physical activity and CP remains to be discussed.

In this study, we used the two-sample MR method to explore the causal relationship between the number of days/week of different levels of physical activity and CP in European, African American, Afro-Caribbean, East Asian, Greater Middle Eastern, and South Asian populations. To the best of our knowledge, our study is the first MR study to investigate the causal relationship between the number of days/week of different levels of physical activity and CP in people of different races. Our results revealed that a higher number of days/week of moderate and vigorous physical activity increased the risk of CP in Europe; however, a higher number of days/week of walking and vigorous physical activity may protect against CP in African American and Afro-Caribbean individuals. The impact of the same level of physical activity on different races may not be the same. Patel’s research showed that even though the proportion of blacks and Hispanics who have sufficient physical activity is similar, there is a significant difference in the incidence of hypertension among them. In addition, the proportion of whites and Asians who have sufficient physical activity is similar, while whites are more than twice as likely as Asians to have coronary artery disease and atherosclerotic cardiovascular disease [29]. Boyer’s study revealed that similar levels of physical activity can reduce all-cause and diabetes-related mortality risk among non-Hispanic whites (and non-Hispanic blacks) but have no effect on Mexican Americans [30,31]. Boyer’s study found significant racial-ethnic differences in several cardiometabolic risk factors among those who had similar levels of physical activity [32]. In addition, ethnicity can impact cortisol, β-endorphin, and allopregnanolone interactions with HPA axis functioning to influence the pain experience [33]. Therefore, the results of our study that the same level of physical activity has different effects on chronic pain in different ethnic groups are also acceptable. Walking is a preventive medicine against weight gain and many chronic health conditions [34]. As one of the health-related physical activities promoted by public health guidelines worldwide, walking prevents functional loss and age-related disability and improves health conditions in people [35]. In our study, the number of days/week with ≥10 min of walking protected against CP in African American and Afro-Caribbean populations, and the results are consistent with other research findings [36]. However, the number of days/week with ≥10 min of walking had little effect on people of other races. The different races may be the main reason for this result. Walking can reduce people’s systolic and diastolic blood pressure responses to stress [37]. It was reported that higher blood pressure was associated with reduced pain in non-Hispanic whites, while there was no relationship between blood pressure and pain in African Americans, demonstrating ethnic differences in endogenous pain regulatory mechanisms [38]. Thus, walking has different effects on chronic pain among people of different races.

The effect of physical activity is generally considered dose dependent, such that a greater amount and higher intensity of physical activity is associated with greater increases in outcomes, for instance, cognitive functions [39] and physical activity-related injuries [40,41]. Injury often leads to chronic pain [42,43]. However, there are also studies with different conclusions. Watts’s research has shown that moderate and vigorous physical activity was related to a lower risk of mortality among an older population [44]. In our study, a higher number of days/week with ≥10 min of moderate physical activity increased the risk of CP in European and South Asian individuals but had little effect on people of other races. A higher number of days/week of ≥10 min of vigorous physical activity increased the risk of CP in Europeans and protected against CP in African Americans and Afro-Caribbeans. The reason for this result may be ethnic differences in endogenous pain regulatory mechanisms, and the types of moderate and vigorous activity are different among different racial populations [29]; thus, a higher number of days/week with ≥10 min of moderate and vigorous physical activity has a negative impact on CP in Europeans. Our results showing that physical activity is beneficial for African Americans and Afro-Caribbean individuals coincide with studies showing that physical activity reduces chronic pain [45,46,47]. However, with the frequent economic and cultural interaction of the world, the migration of people today has produced ancestral variation between populations and on the genome within individuals [48]. In this context, the mixing of human genes may lead to new conclusions regarding the impact of physical activity frequency on CP in different populations in the future. In addition, recent studies have proved that the binomial education in pain neuroscience and therapeutic exercise have a high impact on CP [49,50,51], that is to say, education in pain neuroscience will also influence the role of exercise in CP. In summary, although physical activity is recommended for everyone, it is particularly important for people to adjust the frequency and intensity of physical activity according to their physical condition.

In addition, our study has several limitations. The first limitation is the small sample size of physical activity data from African American, Afro-Caribbean, East Asian, Greater Middle Eastern, and South Asian populations (*n* < 10,000). Second, our study contained several racial populations, which may limit the application of the conclusions to other racial populations. Third, there are potential differences in self-reported measures of physical activity among different populations, which may bias the results. Furthermore, our study lacked detailed information on different levels of physical activity, so we could not perform a subgroup analysis.

## 5. Conclusions

In conclusion, our study demonstrated the associations between the number of days/week with ≥10 min of various categories of physical activity and CP. A higher number of days/week of moderate and vigorous physical activity increased the risk of CP in Europeans; however, a higher number of days/week of walking and vigorous physical activity may protect against CP in African Americans or Afro-Caribbeans. Therefore, different intensity of physical activity may help to reduce CP risk in different ethnic groups. Our findings may provide guidance and a reference for the prevention of CP in people of different races origins from the perspective of physical activity.

## Figures and Tables

**Table 1:** Detailed information on the GWAS datasets used in this MR study.

GWAS ID	Trait	Sample Size	Case	Control	SNPs	Consortium (Author)	Population	Year
ukb-a-506	Number of days/week with ≥10 min of walking	331,654	NA	NA	10,894,596	Neale Lab	European	2017
ukb-e-864-AFR	Number of days/week with ≥10 min of walking	6271	6271	0	15,532,486	Pan-UKB team	African American or Afro-Caribbean	2020
ukb-e-864-EAS	Number of days/week with ≥10 min of walking	2558	2558	0	8,256,758	Pan-UKB team	East Asian	2020
ukb-e-864-MID	Number of days/week with ≥10 min of walking	1508	1508	0	11,867,624	Pan-UKB team	Greater Middle Eastern (Middle Eastern, North African, or Persian)	2020
ukb-e-864-CSA	Number of days/week with ≥10 min of walking	8233	8233	0	9,810,548	Pan-UKB team	South Asian	2020
ukb-a-508	Number of days/week with ≥10 min of moderate physical activity	321,309	NA	NA	10,894,596	Neale Lab	European	2017
ukb-e-884-AFR	Number of days/week with ≥10 min of moderate physical activity	5870	5870	0	15,533,667	Pan-UKB team	African American or Afro-Caribbean	2020
ukb-e-884-EAS	Number of days/week with ≥10 min of moderate physical activity	2402	2402	0	8,263,434	Pan-UKB team	East Asian	2020
ukb-e-884-MID	Number of days/week with ≥10 min of moderate physical activity	1407	1407	0	11,801,052	Pan-UKB team	Greater Middle Eastern (Middle Eastern, North African, or Persian)	2020
ukb-e-884-CSA	Number of days/week with ≥10 min of moderate physical activity	7481	7481	0	9,808,866	Pan-UKB team	South Asian	2020
ukb-a-511	Number of days/week with ≥10 min of vigorous physical activity	321,533	NA	NA	10,894,596	Neale Lab	European	2017
ukb-e-904-AFR	Number of days/week with ≥10 min of vigorous physical activity	5690	5690	0	15,533,580	Pan-UKB team	African American or Afro-Caribbean	2020
ukb-e-904-EAS	Number of days/week with ≥10 min of vigorous physical activity	2365	2365	0	8,264,757	Pan-UKB team	East Asian	2020
ukb-e-904-MID	Number of days/week with ≥10 min of vigorous physical activity	1392	1392	0	11,821,327	Pan-UKB team	Greater Middle Eastern (Middle Eastern, North African, or Persian)	2020
ukb-e-904-CSA	Number of days/week with ≥10 min of vigorous physical activity	7201	7201	0	9,809,619	Pan-UKB team	South Asian	2020
ukb-e-MCP_AFR	Multisite chronic pain	6195	6195	0	15,534,171	Pan-UKB team	African American or Afro-Caribbean	2020

Abbreviations: GWAS ID, GWAS identifier; trait, the content of the dataset; SNPs, single-nucleotide polymorphisms.

**Table 2:** Causal effects of the number of physical activities on MCP in people of different races using different Mendelian randomization (MR) methods and a heterogeneity analysis.

Exposure	Forward MR	β	SE	*p*	OR	95%CI (OR)
Ukb-a-506	MR-Egger	−0.006	0.05448	0.918	0.994	0.894–1.106
	Weighted median	−0.028	0.0554	0.6121	0.972	0.872–1.084
	Inverse-variance weighting	0.022	0.03992	0.5841	1.022	0.945–1.105
	Weighted mode	−0.027	0.0523	0.6059	0.973	0.878–1.078
Ukb-e-864-AFR	MR-Egger	0.019	0.027	0.477	1.019	0.967–1.075
	Weighted median	−0.009	0.015	0.576	0.991	0.962–1.022
	Inverse-variance weighting	−0.021	0.011	0.045	0.979	0.959–1.000
	Weighted mode	0.004	0.040	0.912	1.004	0.929–1.086
Ukb-e-864-EAS	MR-Egger	−0.005	0.015	0.765	0.995	0.966–1.025
	Weighted median	−0.008	0.010	0.376	0.992	0.973–1.010
	Inverse-variance weighting	−0.009	0.007	0.214	0.991	0.978–1.005
	Weighted mode	−0.007	0.011	0.499	0.993	0.972–1.014
Ukb-e-864-MID	MR-Egger	0.020	0.015	0.195	1.020	0.990–1.051
	Weighted median	−0.001	0.008	0.929	0.999	0.983–1.016
	Inverse-variance weighting	−0.001	0.006	0.873	0.999	0.988–1.010
	Weighted mode	0.017	0.017	0.324	1.017	0.984–1.052
Ukb-a-508	MR-Egger	0.202	0.102	0.0499	1.223	1.002–1.495
	Weighted median	0.137	0.070	0.050	1.147	1.000–1.316
	Inverse-variance weighting	0.139	0.045	0.002	1.149	1.052–1.256
	Weighted mode	0.133	0.102	0.194	1.143	0.935–1.396
Ukb-e-884-AFR	MR-Egger	−0.031	0.026	0.228	0.969	0.921–1.019
	Weighted median	−0.005	0.012	0.653	0.995	0.971–1.018
	Inverse-variance weighting	−0.008	0.008	0.334	0.992	0.976–1.008
	Weighted mode	0.001	0.036	0.987	1.001	0.933–1.073
Ukb-e-884-EAS	MR-Egger	−0.009	0.009	0.327	0.991	0.975–1.008
	Weighted median	−0.008	0.007	0.225	0.992	0.979–1.005
	Inverse-variance weighting	−0.008	0.004	0.080	0.992	0.983–1.001
	Weighted mode	−0.008	0.007	0.254	0.992	0.979–1.006
Ukb-e-884-MID	MR-Egger	−0.004	0.012	0.750	0.996	0.973–1.020
	Weighted median	−0.005	0.007	0.465	0.995	0.983–1.008
	Inverse-variance weighting	−0.005	0.004	0.202	0.995	0.987–1.003
	Weighted mode	−0.012	0.011	0.277	0.988	0.967–1.010
Ukb-e-884-CSA	MR-Egger	0.024	0.032	0.453	1.024	0.962–1.091
	Weighted median	0.014	0.019	0.456	1.014	0.978–1.052
	Inverse-variance weighting	0.032	0.012	0.007	1.032	1.009–1.056
	Weighted mode	0.012	0.024	0.602	1.012	0.967–1.060
Ukb-a-511	MR-Egger	0.243	0.207	0.242	1.275	0.850–1.913
	Weighted median	0.147	0.098	0.133	1.158	0.956–1.402
	Inverse-variance weighting	0.217	0.067	0.001	1.243	1.090–1.416
	Weighted mode	0.047	0.171	0.782	1.049	0.750–1.466
Ukb-e-904-AFR	MR-Egger	0.009	0.031	0.780	1.009	0.949–1.072
	Weighted median	−0.031	0.014	0.023	0.969	0.944–0.996
	Inverse-variance weighting	−0.025	0.010	0.010	0.975	0.957–0.994
	Weighted mode	−0.036	0.035	0.297	0.964	0.901–1.032
Ukb-e-904-EAS	MR-Egger	0.004	0.017	0.812	1.004	0.971–1.039
	Weighted median	−0.011	0.012	0.335	0.989	0.967–1.012
	Inverse-variance weighting	0.004	0.007	0.576	1.004	0.990–1.018
	Weighted mode	−0.014	0.013	0.295	0.986	0.960–1.012
Ukb-e-904-MID	MR-Egger	0.000	0.0118	0.993	1.000	0.977–1.023
	Weighted median	0.001	0.007	0.913	1.001	0.987–1.015
	Inverse-variance weighting	0.001	0.005	0.813	1.001	0.991–1.011
	Weighted mode	−0.006	0.014	0.690	0.994	0.967–1.023
Ukb-e-904-CSA	MR-Egger	−0.011	0.028	0.692	0.989	0.937–1.044
	Weighted median	−0.005	0.017	0.792	0.996	0.963–1.029
	Inverse-variance weighting	0.001	0.014	0.956	1.001	0.974–1.028
	Weighted mode	0.0001	0.018	0.993	0.100	0.965–1.035

**Table 3:** Heterogeneity analysis of causal effect estimates for physical activity on MCP.

9	Heterogeneity Statistics		Horizontal Pleiotropy		
	MR-Egger *p* Value	IVW *p* Value	Egger Regression Intercept	Standard Error	Directionality *p* Value
Ukb-a-506	0.108	0.113	0.002	0.003	0.459
Ukb-e-864-AFR	0.937	0.917	−0.009	0.006	0.107
Ukb-e-864-EAS	0.106	0.122	−0.002	0.007	0.749
Ukb-e-864-MID	0.604	0.564	−0.011	0.007	0.146
Ukb-a-508	0.229	0.238	−0.003	0.004	0.496
Ukb-e-884-AFR	0.759	0.760	0.006	0.006	0.344
Ukb-e-884-EAS	0.845	0.836	0.001	0.006	0.926
Ukb-e-884-MID	0.143	0.157	−0.001	0.007	0.902
Ukb-e-884-CSA	0.191	0.215	0.002	0.008	0.800
Ukb-a-511	0.143	0.157	0.001	0.006	0.895
Ukb-e-904-AFR	0.845	0.836	−0.008	0.007	0.252
Ukb-e-904-EAS	0.689	0.726	−0.00004	0.007	0.996
Ukb-e-904-MID	0.013	0.015	0.001	0.007	0.918
Ukb-e-904-CSA	0.004	0.004	0.004	0.008	0.624

## Data Availability

The data used are available from a Genome-Wide Association Studies (GWASs) data resources. Further information can be found on the website https://gwas.mrcieu.ac.uk/, accessed on 16 September 2023.

## Supplementary Materials

The following supporting information can be downloaded at: https://www.mdpi.com/article/10.3390/jpm14010050/s1, Table S1: Characteristics of instrumental variables for the number of days/week of different levels of physical activity.
